# Effective Use of Mobile Electronic Medical Records by Medical Interns in Real Clinical Settings: Mixed Methods Study

**DOI:** 10.2196/23622

**Published:** 2020-12-15

**Authors:** SuJin Kim, Seulji Ku, Taerim Kim, Won Chul Cha, Kwang Yul Jung

**Affiliations:** 1 Department of Digital Health Samsung Advanced Institute for Health Science & Technology Sungkyunkwan University Seoul Republic of Korea; 2 Research Institute for Future Medicine Samsung Medical Center Seoul Republic of Korea; 3 Department of Emergency Medicine Samsung Medical Center Seoul Republic of Korea; 4 Health Information and Strategy Center Samsung Medical Center Seoul Republic of Korea; 5 Department of Emergency Medicine Ewha Womans University Seoul Hospital Seoul Republic of Korea

**Keywords:** mobile health, mobile EMR, intern, workflow, mHealth, electronic medical record, electronic health record, EHR, EMR, efficiency

## Abstract

**Background:**

In South Korea, most graduated medical students undertake a 1-year internship before beginning residency and specialization. Interns usually work in a tertiary hospital and rotate between different, randomly assigned departments to be exposed to different medical specialties. Their jobs are mostly simple and repetitive but are still essential for the patient care process. However, owing to the lack of experience and overwhelming workload, interns at tertiary hospitals in South Korea are usually inefficient, often delaying the entire clinical process. Health care providers have widely adopted mobile electronic medical records (mEMRs) as they have been shown to improve workflow efficiency.

**Objective:**

This study investigates the association between the frequency of mEMR usage and the clinical task completion interval time among interns in a tertiary hospital.

**Methods:**

This mixed methods study was conducted at the Samsung Medical Center, Seoul, South Korea. Interns who worked at the Samsung Medical Center from March 2018 to February 2019 were included. The hospital electronic medical record (EMR) system known as DARWIN (Data Analysis and Research Window for Integrated kNowledge) was launched with PC and mobile. Both versions are actively used in hospitals by personnel in various positions. We collected the log data from the mEMR server and the intern clinical task time-series data from the EMR server. Interns can manage the process of identifying patients, assigning the clinical task, finishing the requested clinical intern tasks, etc, through the use of the mEMR system. We compared the clinical task completion interval among 4 groups of interns divided by the mEMR frequency quantile. Then, System Usability Score (SUS) questionnaires and semistructured interviews were conducted.

**Results:**

The regular mEMR users were defined as those who logged in more than once a day on average and used the mEMR until the level after login. Among a total of 87 interns, 84 used the mEMR to verify the requested clinical tasks. The most frequently used item was “Intern task list.” Analysis of the 4 intern groups revealed an inverse relationship between the median time of the task completion interval and the frequency of mEMR use. Correlation analysis showed that the intern task completion time interval had a significant inverse relationship with the individual frequency of mEMR usage (coefficient=-0.27; 95% CI -0.46 to -0.04; *P*=.02). In the additional survey, the mean SUS value was 81.67, which supported the results of the data analysis.

**Conclusions:**

Our findings suggest that frequent mEMR use is associated with improved work efficiency in hospital interns with good usability of the mEMR. Such finding supports the idea that the use of mEMR improves the effectiveness and workflow efficiency of interns working in hospitals and, more generally, in the context of health care.

## Introduction

### Background and Significance

Professionals of various occupations, such as doctors, nurse pharmacists, and other supporters, provide patient care in hospitals. In most tertiary hospitals in South Korea, prescribers, such as specialists and residents, determine the appropriate care plan and use computerized provider order entry (CPOE) to order prescriptions. Then a nurse executes the order or passes it on to interns or other supporters. This computerized linear workflow benefits workflow and patient safety [1,2]; however, when overloaded, it inevitably results in inefficiency and delay [3].

Internship is the transition period between being a medical student and becoming a specialty doctor [4]. In South Korea, most graduated medical students undertake a 1-year internship with their physician's license before beginning their specialty resident course [5]. Interns usually work in a tertiary hospital, where they rotate monthly among different, randomly assigned departments to be exposed to different medical specialties. Their jobs are mostly simple and repetitive, but they are essential for the patient care process. These jobs include simple procedures (eg, catheterization, biopsies, monitoring, and sampling), documentation (eg, getting consent forms for radiology or procedures), and prescriptions that do not affect patient care plans (eg, meal changes, simple dressing) [6].

The lack of experience and the workload of interns at tertiary hospitals, where patient needs are substantial and often overwhelming, make interns inevitably susceptible to inefficiency and fatigue [7]. Such inefficiency can halt the entire clinical process and expose patients to the risk of errors. This can ultimately have an adverse influence on patient care and safety [8].

### Mobile Electronic Medical Records for Health Care Providers

Before the use of smartphones, interns usually received notifications pertaining to their jobs through various mediums (depending on the policies of each hospital), such as pagers, phone calls, or beepers for SMS texting exclusively used in the hospital communication system [9]. With the widespread use of smartphones, health care professionals have widely adopted mobile electronic medical records (mEMRs) [10-12]. The mEMR has been shown to improve the efficiency and effectiveness of hospital workflow in previous studies [13,14]. However, none of the previous studies evaluated the time efficiency of interns’ job achievement in a clinical setting using mEMRs.

### Study Objective

This study aims to determine the association between interns' clinical task completion time interval and the frequency of mEMR usage.

## Methods

### Study Setting

This mixed methods study was conducted at the Samsung Medical Center, Seoul, South Korea. We targeted and analyzed interns who worked at the Samsung Medical Center from March 2018 to February 2019. In South Korea, from the month of March to the following February, interns rotate between various medical departments. To examine the association between mEMR usage and intern performance, we collected the log data from the mEMR server and the intern clinical task time-series data from Samsung Medical Center's Electronic Medical Record (EMR). The study protocol was reviewed and approved by the institutional review board (IRB) of Samsung Medical Center (IRB No. SMC 2019-09-122-001).

### Mobile Electronic Medical Records

In July 2016, the hospital EMR system known as DARWIN (Data Analysis and Research Window for Integrated kNowledge) was launched. DARWIN has both PC and mobile versions. DARWIN is actively used in hospitals, and its mobile version is used by hospital personnel in various positions. Mobile DARWIN (mDARWIN) includes a main menu, list-level features, and patient-level features. After login into the mDARWIN, users can select a list-level feature on the first screen from 8-9 main menus.

### Interns' Clinical Task Implementation Process

**Figure 1:**
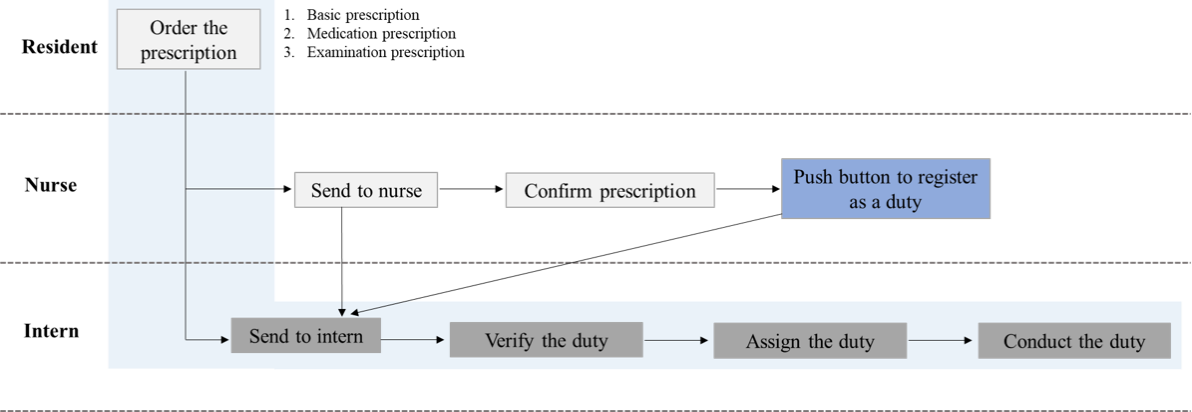
Flowchart of an intern’s clinical task process.

**Figure 2:**
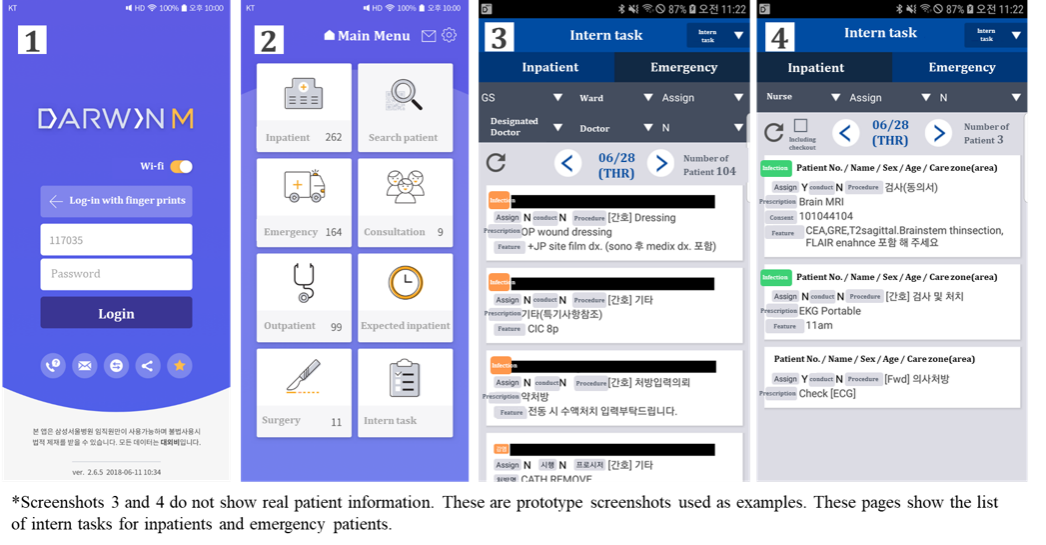
Screenshot of intern tasks from Mobile DARWIN (mDARWIN).

There are 3 types of prescriptions that prescribers such as specialists or residents issue: (1) basic prescription (eg, vital-sign check term, input and output check term, meal, or simple daily care service for postoperation patients, (2) medication prescription, and (3) examination prescription. These prescriptions have associated tasks that are performed by health care providers. With the exception of the tagging of prescriptions (which is performed by nurses), most clinical tasks are performed by interns. When interns receive an alarm about a new task on their mobile device, they verify the clinical task and self-assign the prescriptions to themselves. Then, they conduct the clinical task according to the instruction (Figure 1). Interns can manage this process (ie, identify, assign, and mark the task as complete after finishing the requested clinical task) through the mDARWIN mEMR (Figure 2).

### Outcome Measures and Sensitivity Analysis

The primary outcome was the comparison of the time interval to complete the intern tasks after dividing the interns into 4 groups based on the quantile of the frequency of mEMR usage. The definition of the task completion interval time was set from the time the task was requested to the task completion check time. For sensitivity analysis, we verified the correlation between the frequency of mEMR usage and the median time of interval to complete the intern's tasks individually. Subsequently, System Usability Score (SUS) questionnaires were administered and analyzed.

### Survey

To investigate the feasibility of the mEMR in a clinical environment, SUS questionnaires were administered to interns [15]. Interns were recruited through a notice posted on the bulletin board in the hospital for 2 months. In addition to the survey, interns were also interviewed at the same time. The survey included 10 questions, scored using a 5-point Likert scale (1=strongly disagree, 5=strongly agree). The SUS calculation formula is as follows:

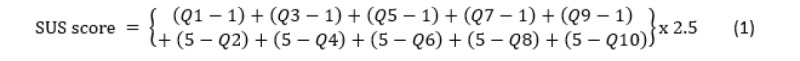



During the interview, interns were asked questions such as when they mostly used the mEMR, which list they searched the most, where they mostly used the mEMR, and whether mDARWIN helps with their tasks. The interviews were semistructured.

### Statistical Analysis

We investigated the log data of interns' mEMRs during the study period. We compared the task completion time interval among the 4 groups using statistical analysis. We compared the task completion interval's median time and 95% confidence interval between the 4 groups.

For sensitivity analysis, we evaluated the correlation between the frequency of log data of interns' mEMR and the individual task completion time interval of interns using the Pearson product-moment correlation coefficient test. *P* values of <.05 were considered statistically significant. All data analyses were performed using R software (version 3.4.2; R Project for Statistical Computing).

## Results

### Characteristics of the Subjects

In total, 87 interns performed intern tasks during the study period. A total of 1,081,413 tasks were performed by these interns. Of the 87 interns, 84 regularly used the mEMR and were included in the analysis. However, 3 interns were excluded because 2 had not used mEMR at all and 1 had a total of only 4 log records during the study period; thus, they were considered nonusers. In this context, regular mEMR users were defined as those who logged in more than once a day on average and used the mEMR until the next level after login. Table 1 shows the intern information included in the study and the clinical tasks they received.

**Table 1:** Information about the study subjects during the study period (n=87).

Participant characteristics			Values, n (%)
**Medical interns (n=87)**			
	mEMR^a^ users		84 (97)
	Non-mEMR^a^ users		3 (3)
**Total intern clinical tasks performed (n=1,081,413)**			
	**By location**		
		Inpatient	940,338 (87.00)
		Outpatient	1336 (0.10)
		Emergency	139,739 (12.90)
	**By department**		
		Medical part	462,018 (42.70)
		Surgical part	478,242 (44.20)
		Other hospital-based part	141,153 (13.10)
	**By procedure category**		
		Request order transcription (from nurse)^b^	348,805 (32.30)
		Request order transcription (from doctor) ^b^	163,886 (15.20)
		Diagnostic test consent form	170,542 (15.80)
		Wound dressing	134,503 (12.40)
		Diagnostic test	94,596 (8.70)
		Diagnostic test and treatment	30,521 (2.80)
		Catheter tube insertion	16,058 (1.50)
		Administrative paperwork	14,533 (1.30)
		Irrigation	14,305 (1.30)
		Influenza exam	5938 (0.50)
		Enema	3245 (0.30)
		Writing slip	2733 (0.30)
		Inject medicine	959 (0.10)
		Other	80,789 (7.50)

^a^mEMR: mobile electronic medical records.

^b^South Korea's medical system adopts a fee-for-service model for medical service. As the prescription order can only be authorized by a doctor, this category is in relation to the prescription after the act of the nurse or doctor.

### Log Data Analysis

During the study period, 489,444 mEMR logs were created by interns. Interns used a total of 43 items within the mEMR, as shown in Multimedia Appendix 1. From the 489,444 logs, 67,147 logs were made in a list-level feature. Among these records, “Intern task list” topped the list with 39,506 tasks. This was followed by “My patient list” and “Surgery history list,” as shown in Table 2.

**Table 2:** Total number of logs with a list-level feature (n=67,147).

No.	List	Frequency, n (%)
1	Intern task list	39,506 (58.80)
2	My patient list	13,685 (20.40)
3	Surgery history list	8,545 (12.70)
4	Inpatient list	3,963 (5.90)
5	Emergency patient list	663 (1.00)
6	Integrated-view EMR^a^ list	241 (0.40)
7	Outpatient list	137 (0.20)
8	Consultation list	110 (0.20)
9	Expected inpatient list	78 (0.10)
10	Scheduled surgery list	46 (0.10)
11	Patient search (through patient ID)	15 (0.00)
12	Other	158 (0.20)

^a^EMR: electronic medical record.

### Statistical Outcomes

**Figure 3:**
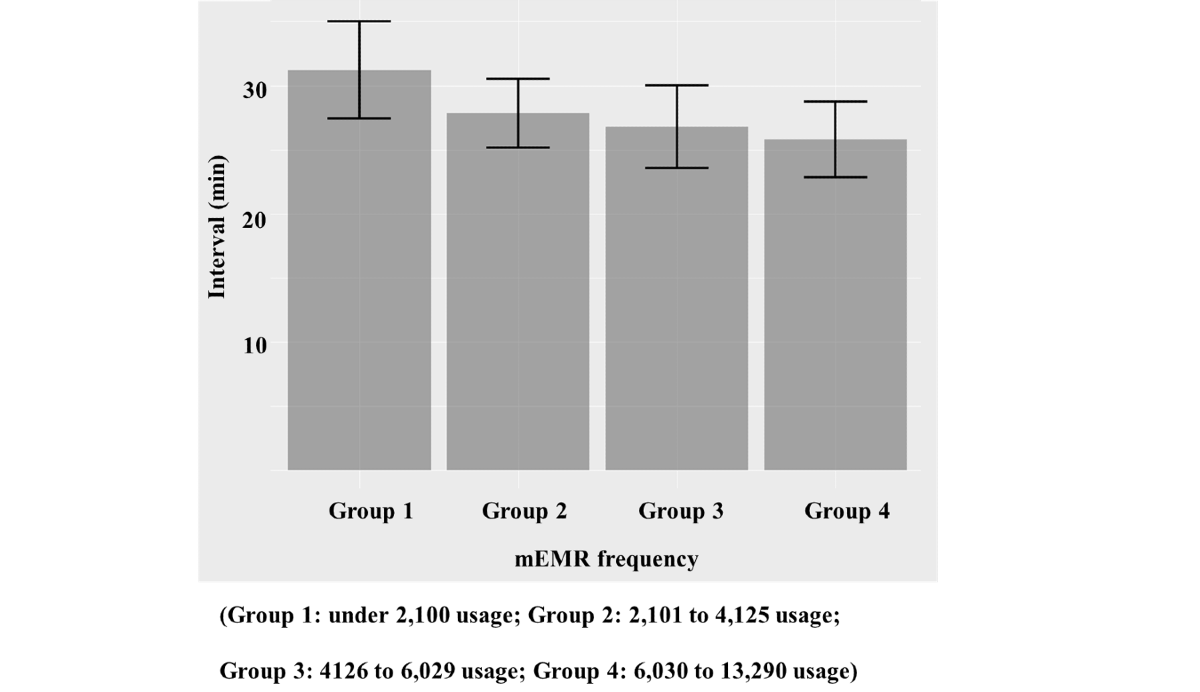
Task completion time interval and frequency of mobile electronic medical record (mEMR) usage among 4 intern groups divided by quantile of mEMR usage.

The comparison of clinical task completion interval consistently showed an inverse relationship between the median time of completion interval and the frequency of mEMR usage (Figure 3). The frequent mEMR user group took a shorter time to complete the requested tasks. Sensitivity analysis with the Pearson product-moment correlation coefficient showed that the intern task completion interval time had a significant inverse relationship with individual frequency of mEMR usage (coefficient -0.27; 95% CI -0.46 to -0.04; *P*=.02). Using the mEMR once reduced the task completion time by approximately 16 seconds (*P*=.02).

### SUS Survey Outcome

**Figure 4:**
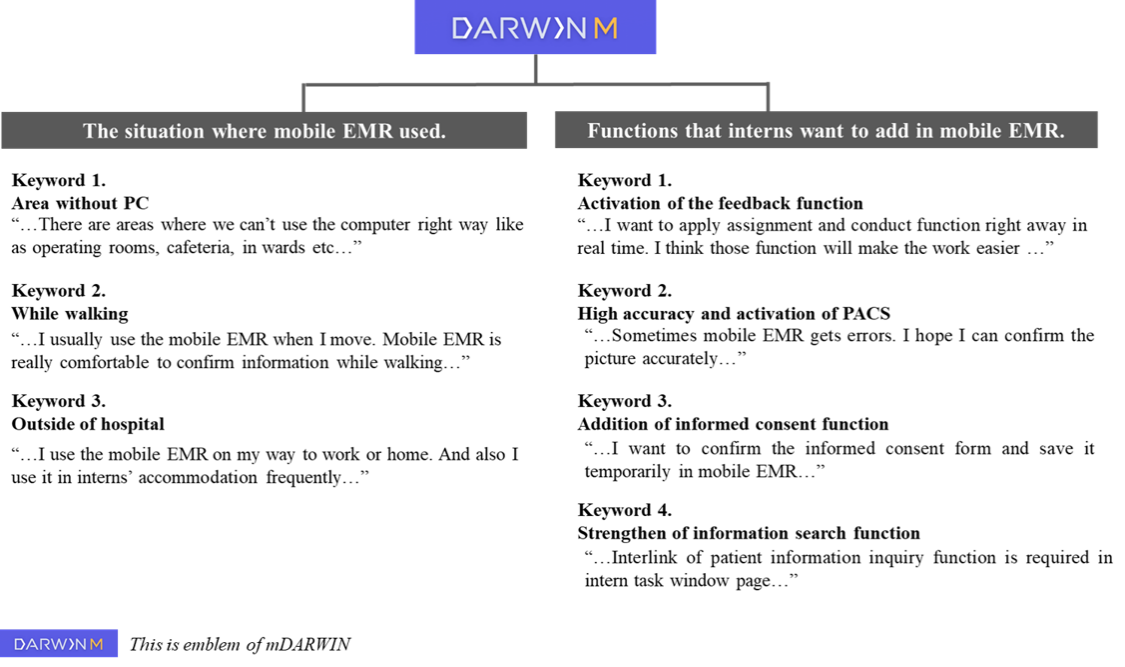
Summary of the intern interviews about mobile electronic medical records (EMR).

A total of 15 interns completed the SUS survey from December 2019 to January 2020. The mean SUS value for the intern clinical task item in the mEMR was 81.67 (Table 3). The interview and survey were conducted at the same time. Figure 4 shows the key points in the interview that may be useful for future research.

**Table 3:** System Usability Score (SUS) survey assessing an intern task item in the mobile electronic medical record (mEMR; n=15). The mean value of the 5-point Likert-scale responses was 3.1 (SD 1.6), and the mean SUS value was 81.67 (SD 9.4).

No.	Question	Response, mean (SD)^a^
1	I think I use (intern task) frequently through mDARWIN^b^.	4.7 (0.6)
2	I found that using (intern task) through mDARWIN^b^ is unnecessarily complex.	1.4 (0.5)
3	I thought that using (intern task) in mDARWIN^b^ was easy.	4.5 (0.5)
4	I think technical support is needed to use (intern task) in mDARWIN^b^.	2.7 (1.4)
5	I found that (intern task) in mDARWIN^b^ was well integrated.	3.9 (1.0)
6	I thought there was too much inconsistency with (intern task) in mDARWIN^b^_._	1.7 (0.7)
7	I would imagine that most people would learn to use (intern task) through mDARWIN^b^ very quickly and easily.	4.7 (0.5)
8	I found that using (intern task) in mDARWIN^b^ is very cumbersome.	1.7 (0.9)
9	I felt very confident using (intern task) through mDARWIN^b^.	4.0 (0.9)
10	I needed to learn many things before I could get going with (intern task) through mDARWIN^b^.	1.5 (0.6)

^a^Responses were scored using a 5-point Likert scale (1=strongly disagree, 5=strongly agree).

^b^mDARWIN: mobile Data Analysis and Research Window for Integrated kNowledge.

## Discussion

### Principal Findings

This study investigated the correlation between the frequency of mEMR usage and the intern task time interval based on mEMR log data and EMR timestamp data. Most interns use mEMR and the task completion time was shorter for interns who used mEMR more frequently. This suggests that mEMR use could effectively enhance hospital workflow time, leading to a faster response in real practice. This result supports the findings of previous studies that indicate that the mEMR is linked to improved workflow efficiency in hospitals by enabling faster responses [13,16,17].

In addition to log data analysis, we also interviewed interns to assess the use of the mEMR for job execution. All the interns who participated in the survey and interview were actively using the mEMR. The mean SUS value was >80, implying that the system is well utilized by the user [18]. Doctors tend to underestimate the various positive workflow effects of mEMR usage [14]; as such, our results are interesting and valuable enough to analyze motivation. We assume that the obvious and dominant benefit of mEMRs in terms of convenience and time efficiency would make all interns maximize the use of the mEMR compared to other systems such as computers and telephones. Further in-depth surveying and analysis can help increase mEMR usage among hospital health care providers. Our study shows that mEMR use offers both quantitative and qualitative strengths for intern job performance.

### Comparison with Prior Work

Studies aiming to investigate the effects of mobile device use among health care providers in hospitals have proven their efficiency via surveys [14,19,20] and in simulations [21,22], and they have shown to be effective in limited spaces such as surgical rooms [23] and emergency departments [24]. However, there are limited quantitative studies assessing the efficiency of mEMR use in clinical practice. Our study results provide further evidence of the efficiency of mEMRs and suggest extending their use to other professionals with relatively similar daily tasks, such as physician assistants (PAs) who are responsible for clinical prescriptions in tertiary hospitals or nurses who are similarly overloaded with work. Further, the use of mEMRs by PAs or nurses would improve workflow efficiency, and ultimately, patient care and patient safety [25].

In recent times, quick response code technology reduces time and errors in patient identification during patient care and procedures [26-28]. Further, the closed-loop medication system, which integrates the barcode medication system and CPOE technology with automated dispensing technology (robots/units), prevents the adverse effects of medication due to administration errors [29]. Future efforts should be directed at combining mEMR use with these technologies to simultaneously achieve efficient workflows and patient safety.

### Limitations

This study has some limitations. First, as this study was performed at a single center, the results have limited generalizability. Further, given that there are different job allocations for each occupation depending on the hospital, its feasibility and usability need to be validated in other institutions and environments.

Second, we could not identify the causality of log data as we analyzed the entire log dataset. There is no consideration for context or order between log data and interns’ jobs. Although the entire log was sufficient to achieve the study aim, a further observational study using the small cut log of mEMR is needed to analyze the association between behavior and mEMR usage.

Third, we did not consider the priority of specific jobs when assessing performance. Jobs related to emergency situations need to be prioritized over others that can be completed after the emergency situation. However, this study aimed to investigate the general trend of frequent mEMR users and not to compare nonfrequent and frequent users to assess the efficiency of the mEMR. Furthermore, the log data was large enough to distinguish between situations.

### Conclusions

By retrospectively analyzing the mEMR log data of hospital interns, this study revealed that more frequent use of the mEMR led to quicker completion of intern jobs. This finding implies the effectiveness of mEMR use for the workflow of interns in hospitals. We used a SUS survey to examine the usability of mEMR, and the survey concluded that the mEMR has a good usability.

## Appendix

Multimedia Appendix 1 Items on the mEMR menu.

## Abbreviations

CPOE: computerized provider order entry
DARWIN: Data Analysis and Research Window for Integrated kNowledge
EMR: electronic medical record
mDARWIN: mobile Data Analysis and Research Window for Integrated kNowledge
mEMR: mobile electronic medical record
SUS: System Usability Score
