# Supplementary material for: Effective Use of Mobile Electronic Medical Records by Medical Interns in Real Clinical Settings: Mixed Methods Study
Source: JMIR Mhealth Uhealth. 2020 Dec 15;8(12):e23622. doi: 10.2196/23622 (PMC7772071; doi:10.2196/23622)
Supplement: Multimedia Appendix 1 [file mhealth_v8i12e23622_app1.pdf]

Multimedia Appendix 1. Items on the mEMR menu

No.	Contents (items)
**1**	Login
**2**	Patient list
**3**	OP history
**4**	OP schedule
**5**	Patient information
**6**	Slip list (based on exam date)
**7**	OP ledger list
**8**	Consultation list
**9**	EMR access right
**10**	Integrated EMR list
**11**	Integrated EMR data
**12**	Order (medicine)
**13**	Order (operation fee)
**14**	PACS list
**15**	Slip list (based on order date)
**16**	Exam result (based on exam date)
**17**	Exam result (based on order date)
**18**	OPD patient list
**19**	CIS image list
**20**	CIS image
**21**	ED patient list
**22**	Vital sign
**23**	Admission schedule
**24**	Note
**25**	Emergency prescription (medicine)
**26**	Emergency nursing report
**27**	Input/output
**28**	Text result
**29**	Consultation request/response
**30**	Infection information
**31**	OPD prescription (medicine)
**32**	Dialysis patient list
**33**	Order (examination) list
**34**	Intern task list

**35**	Slip list (based on OPD order date)
**36**	Exam result (based on OPD order date)
**37**	Slip list (based on OPD exam date)
**38**	NFC ED patient list
**39**	Tube drain amount
**40**	Patient search (name)
**41**	Patient search (ID)
**42**	My patient count badge
**43**	Input/output

OP: operation, **EMR:** Electronic Medical Records, **PACS:** Picture Archiving Communications System, **OPD:** outpatient department, **ED**: emergency department, **NFC:** nearfield communication
